# Dental Tissue-Derived Mesenchymal Stem Cells Modulate Mitochondrial and *OPG/RANKL* Signaling in Obesity-Associated Osteoporosis Under Estrogen-Deficient and Intact Conditions

**DOI:** 10.3390/biomedicines14061320

**Published:** 2026-06-10

**Authors:** Saet-Byul Kim, Chae-Yeon Hong, Won-Jae Lee, Hyeon-Jeong Lee, Chan-Hee Jo, Seo-Yoon Kang, Sanghyeon Park, Yeung Bae Jin, Tae-Sung Hwang, Jaemin Kim, Yong-ho Choe, Sung-Lim Lee

**Affiliations:** 1 Bovivet, Gumi 39133, Republic of Korea; bovivet.sbkim@gmail.com; 2 College of Veterinary Medicine, Gyeongsang National University, Jinju 52828, Republic of Koreach_jo@gnu.ac.kr (C.-H.J.);; 3 College of Veterinary Medicine, Kyungpook National University, Daegu 41566, Republic of Korea; 4 Strategy & Planning Bureau, Daegu-Gyeongbuk Medical Innovation Foundation, Daegu 41061, Republic of Korea; 5 Division of Applied Life Science, Gyeongsang National University, Jinju 52828, Republic of Korea; jmkim85@gnu.ac.kr; 6 Institute of Agriculture and Life Sciences, Gyeongsang National University, Jinju 52828, Republic of Korea; 7 Institute of Animal Medicine, Gyeongsang National University, Jinju 52828, Republic of Korea; 8 Research Institute of Life Sciences, Gyeongsang National University, Jinju 52828, Republic of Korea

**Keywords:** mesenchymal stem cells, obesity-associated osteoporosis, estrogen receptor signaling, OPG/RANKL axis, mitochondrial biogenesis

## Abstract

**Background/Objectives**: Obesity and menopause are major determinants of skeletal deterioration; however, their combined effects on bone remodeling and associated cellular bioenergetics remain incompletely understood. This study aimed to determine whether obesity induces osteoporotic alterations under both estrogen-replete and estrogen-deficient conditions and to evaluate the therapeutic potential of dental tissue-derived mesenchymal stem cells (D-MSCs). **Methods**: Female mice were subjected to ovariectomy (OVX) and/or high-fat diet (HFD) feeding for 16 weeks to establish obesity-associated osteoporosis models. D-MSCs were administered intraperitoneally at defined intervals. Body weight and serum leptin levels were measured to assess metabolic status. Femoral tissues were analyzed by quantitative real-time PCR for estrogen receptors (*ERα*, *ERβ*), inflammatory markers (*Il-1β*, *Tnf-α*), mitochondrial regulators (*Pgc1α*, *Pgc1β*), and the *OPG/RANKL* ratio. Histological analysis was performed to evaluate bone marrow adiposity. **Results**: HFD significantly increased body weight and serum leptin levels in both intact and OVX mice. Obesity was associated with reduced expression of *ERα* and *ERβ*, decreased *Pgc1α* levels, and a lower *OPG/RANKL* ratio, accompanied by increased *Il-1β*, *Tnf-α*, and *Pgc1β* expression. D-MSC administration attenuated body weight gain and reduced leptin levels, particularly in OVX mice. In femoral tissue, D-MSC treatment restored estrogen receptor expression, increased *Pgc1α*, decreased *Pgc1β*, and normalized the *OPG/RANKL* ratio. In addition, inflammatory marker expression and bone marrow adiposity were reduced following MSC administration. **Conclusions**: Obesity induces bone remodeling dysregulation under both intact and estrogen-deficient conditions, characterized by altered estrogen signaling, inflammatory activation, and mitochondrial imbalance. D-MSC administration was associated with partial restoration of these alterations, suggesting a potential role in modulating metabolic and skeletal homeostasis in obesity-associated bone loss.

## 1. Introduction

Obesity and menopause are two major and increasingly prevalent conditions that profoundly influence skeletal homeostasis. The causal relationship between menopause and osteoporosis is well established, primarily driven by estrogen deficiency-mediated imbalance in bone remodeling [1,2,3]. In contrast, the role of obesity in bone metabolism remains controversial. While increased body mass has traditionally been considered protective due to enhanced mechanical loading and peripheral estrogen production, accumulating evidence indicates that obesity is associated with chronic low-grade inflammation and metabolic dysfunction, both of which negatively affect bone remodeling [4,5,6]. Consequently, whether obesity mitigates or exacerbates osteoporosis—particularly under different estrogenic conditions—remains unresolved.

Bone remodeling is a tightly regulated process governed by the coordinated activity of osteoblasts and osteoclasts, ensuring skeletal integrity and mineral homeostasis [7]. Estrogen plays a central role in this process by modulating the osteoprotegerin (*OPG*)/receptor activator of nuclear factor-κB ligand (*RANKL*) axis. Under physiological conditions, estrogen suppresses *RANKL* expression while promoting OPG production, thereby limiting osteoclast differentiation and activity [8,9]. In postmenopausal states, estrogen deficiency disrupts this balance, shifting the *OPG/RANKL* ratio toward osteoclast activation and increased bone resorption [10,11,12]. However, obesity introduces additional metabolic perturbations—including adipokine dysregulation, inflammatory cytokine activation, and altered cellular energy metabolism—that may further exacerbate skeletal imbalance beyond classical hormonal effects.

Emerging evidence highlights mitochondrial function as a critical determinant of bone remodeling. Osteoblast differentiation and function are highly dependent on mitochondrial biogenesis and oxidative metabolism, whereas osteoclast activity is associated with distinct bioenergetic adaptations. Key regulators such as peroxisome proliferator-activated receptor gamma coactivator-1 alpha (*Pgc1α*) and *Pgc1β* have been implicated in osteoblast and osteoclast regulation, respectively [13,14]. Chronic metabolic stress, as observed in obesity, may impair mitochondrial homeostasis, thereby disrupting osteoblastogenesis while promoting osteoclast-mediated bone resorption. Despite these insights, the interplay between obesity, estrogen status, mitochondrial regulation, and inflammatory signaling within bone tissue remains poorly defined.

Current therapeutic strategies for osteoporosis, including bisphosphonates and hormone replacement therapy, primarily focus on inhibiting bone resorption but do not restore bone regenerative capacity and may be associated with adverse effects [15,16,17,18,19]. Therefore, there is a critical need for therapeutic approaches that not only suppress pathological bone loss but also actively restore bone remodeling through coordinated regulation of inflammation, mitochondrial function, and osteogenic activity.

Mesenchymal stem cells (MSCs) have emerged as promising candidates for regenerative therapy due to their multilineage differentiation potential, paracrine signaling capacity, and immunomodulatory properties [20]. In the context of skeletal disorders, MSCs are particularly attractive because they can directly and indirectly influence bone remodeling. MSCs secrete osteogenic factors such as transforming growth factor-β (*TGFβ*), insulin-like growth factor-1 (*IGF1*), and fibroblast growth factor (*FGF*), which promote osteoblast differentiation and matrix formation, while also releasing cytokines that modulate the bone microenvironment [15]. In addition, MSCs can suppress inflammatory signaling and contribute to tissue homeostasis through immunomodulatory mechanisms [21]. Notably, recent studies have demonstrated that MSCs can transfer functional mitochondria to damaged cells, thereby restoring cellular bioenergetics and viability [22]. These properties suggest that MSCs may exert therapeutic effects not only through differentiation and paracrine activity but also through metabolic reprogramming of target tissues.

Among MSC sources, dental tissue-derived MSCs (D-MSCs) represent an accessible and ethically favorable population with high osteogenic potential [23]. D-MSCs have been reported to exhibit enhanced expression of osteogenesis-related genes compared with bone marrow-derived MSCs, indicating their suitability for bone regenerative applications [24]. However, their therapeutic efficacy has not been evaluated in a model that simultaneously incorporates obesity-induced metabolic stress and estrogen deficiency, nor has their impact on mitochondrial regulation within bone tissue been systematically investigated.

In the present study, we aimed to determine whether obesity induces osteoporotic alterations under both intact and ovariectomized conditions and to evaluate the therapeutic potential of human D-MSCs in this context. By integrating analyses of estrogen receptor signaling, inflammatory mediators, mitochondrial regulators (*Pgc1α* and *Pgc1β*), and the *OPG/RANKL* axis, this study seeks to elucidate the mechanistic links between metabolic dysfunction and skeletal deterioration. Furthermore, we investigated whether systemic MSC administration can restore bone remodeling homeostasis through coordinated modulation of inflammatory and mitochondrial pathways under both estrogen-replete and estrogen-deficient conditions.

## 2. Materials and Methods

Chemicals and media: All chemicals were purchased from Sigma-Aldrich^®^ (St. Louis, MO, USA) and culture media were purchased from Gibco Life Technologies (Gaithersburg, MD, USA), unless otherwise specified.

Isolation and cultivation of dental tissue-derived MSCs: Dental follicle tissues were harvested from extracted teeth as previously described [25,26]. Briefly, dental follicle tissues were minced into 1–3 mm^2^ explants using fine scissors and enzymatically digested in D-PBS containing 1 mg/mL collagenase type I at 37 °C with gentle agitation for 40 min. Following digestion, tissues were washed with D-PBS and centrifuged at 500× *g* for 5 min. The digested tissues were then mechanically dissociated and filtered through a 40 μm cell strainer (BD Falcon, Franklin Lakes, NJ, USA) to obtain single-cell populations. Cells (5 × 10^5^) were seeded in 4 mL of Advanced Dulbecco’s Modified Eagle’s Medium (A-DMEM) supplemented with 10% fetal bovine serum (FBS), 1% L-glutamine (GlutaMAX™), 100 IU/mL penicillin, and 100 μg/mL streptomycin. Cells were maintained at 37 °C in a humidified atmosphere containing 5% CO_2_ in air. The culture medium was replaced every 3 days.

Flow cytometry analysis: MSCs at passage 3 were analyzed for the expression of cell surface markers using flow cytometry (BD FACSVerse™, Becton Dickson, NJ, USA). Cells were detached using 0.25% trypsin–EDTA and fixed with 3.7% formaldehyde solution for at least 30 min. Fixed cells were incubated for 30 min at 4 °C with fluorescence-conjugated antibodies at 1:100 dilution as follows: FITC-conjugated anti-CD34; FITC-conjugated anti-CD45; FITC-conjugated anti-CD44; FITC-conjugated anti-CD90; APC-conjugated anti-CD73; and APC-conjugated anti-CD105 (BD Pharmingen™, San Diego, CA, USA). Flow cytometry data were analyzed using FlowJo v10 software. All antibodies used are listed in Table 1.

RNA extraction and quantitative real-time PCR: Total RNA was extracted using the easy-spin™ Total RNA Extraction Kit (iNtRON Biotechnology, Seongnam, Republic of Korea). RNA concentration was measured using a NanoDrop spectrophotometer. Complementary DNA was synthesized from 500 ng RNA using HiSenScript™ RT PreMix (iNtRON Biotechnology). Quantitative PCR was performed using Rotor-Gene Q (QIAGEN, Hilden, Germany) with RealMOD™ Green AP 5× qPCR mix (iNtRON Biotechnology). Cycling conditions were: 95 °C for 12 min, followed by 40 cycles at 95 °C for 15 s, 60 °C for 25 s, and 72 °C for 25 s. Expression of estrogen receptors, inflammatory genes (*Il-1β* and *Tnf-α*), mitochondrial regulators (*Pgc1α* and *Pgc1β*), and *OPG/RANKL* was analyzed. *YWHAZ* (human) and *TBP* (mouse) were used as internal controls. Relative expression levels were calculated using the ΔΔCt method. All reactions were performed in triplicate. All primer sequences are listed in Table 2a.

*In vitro* differentiation into mesenchymal lineages: Passage 4–5 MSCs were induced toward osteogenic, adipogenic, and chondrogenic lineages for 21 days. Osteogenic differentiation was performed in DMEM supplemented with 10% FBS, 0.1 μM dexamethasone, 0.2 mM ascorbic acid-2-phosphate, and 10 mM β-glycerophosphate. Mineralization was assessed by Alizarin Red S and Von Kossa staining. Adipogenic differentiation medium contained 10% FBS, 1 μM dexamethasone, 10 μM insulin, and 100 μM indomethacin. Lipid accumulation was detected using Oil Red O staining. Chondrogenic differentiation was induced using StemPro^®^ Chondrogenesis differentiation medium and evaluated by Alcian Blue staining. Stained cells were examined under a phase-contrast microscope. Expression of lineage-specific genes was analyzed by qRT-PCR, and primer sequences are listed in Table 2b.

Preparation of MSCs for injection: MSCs at passage 4–5 were detached using 0.25% trypsin–EDTA, washed with DPBS, and counted using a hemocytometer. A total of 1 × 10^6^ cells were resuspended in 200 μL cold DPBS for intraperitoneal injection.

Animal model of obesity-induced osteoporosis: Eight-week-old female C57BL/6J mice (17–20 g) were obtained from Central Lab Animal Inc. (Seoul, Republic of Korea). Animals were housed under controlled conditions (25 ± 2 °C, 30–40% humidity, 12 h light/dark cycle) with ad libitum access to food and water. Obesity was induced using a 60 kcal% high-fat diet (Research Diets, Inc., New Brunswick, NJ, USA) administered for 16 weeks.

Mice were randomly assigned to six groups:

  (i) *Intact ND* (n = 5): normal diet for 16 weeks.
 (ii) *Intact HFD* (n = 6): 60 kcal% fat diet for 16 weeks.
(iii) *Intact HFD + MSCi* (n = 6): 60 kcal% fat diet for 16 weeks and i.p. injection of MSCs at weeks 5, 7, 9, 11, 13, and 15 after HFD feeding.
(iv) *OVX-ND* (n = 6): ovariectomy and normal diet for 16 weeks.
 (v) *OVX-HFD* (n = 6): ovariectomy and 60 kcal% fat diet for 16 weeks.
(vi) *OVX-HFD + MSCi* (n = 6): ovariectomy and 60 kcal% fat diet for 16 weeks and i.p. injection of MSCs at weeks 5, 7, 9, 11, 13, and 15 after HFD feeding.

Ovariectomy (OVX) was performed to induce estrogen deficiency. For ovariectomy, mice were anesthetized by intraperitoneal injection of tiletamine–zolazepam (Zoletil^®^; Virbac, Carros, France) and xylazine hydrochloride (Rompun^®^; Bayer, Leverkusen, Germany). After surgery, mice were monitored daily for recovery, wound healing, mobility, and general health condition throughout the postoperative period. One week after surgery, mice were fed either a normal diet or a 60 kcal% high-fat diet for 16 weeks. MSC-treated groups received intraperitoneal injections of 1 × 10^6^ MSCs at weeks 5, 7, 9, 11, 13, and 15 after initiation of HFD feeding. Body weight was recorded weekly. After 16 weeks, mice were sacrificed. Blood samples were collected by cardiac puncture. Femurs were harvested, weighed, and processed for molecular and histological analyses. All procedures were approved by the Institutional Animal Care and Use Committee of Gyeongsang National University (GNU-181210-M0063).

ELISA: Serum was isolated by centrifugation at 400× *g* for 10 min and stored at −80 °C. Serum leptin levels were measured using a commercial ELISA kit (Abcam, Cambridge, UK) according to the manufacturer’s protocol.

Histology: Femurs were fixed in 4% formaldehyde, decalcified in 0.05 M EDTA for 4 weeks, embedded in paraffin, and sectioned at 5 μm. Sections were stained with hematoxylin and eosin (H&E) and examined under a light microscope Nikon Eclipse 80i( Nikon Corporation, Tokyo, Japan). Images were captured using a digital imaging system. For quantification of marrow lipid accumulation, H&E-stained femoral sections were analyzed using ImageJ software (version 1.54k, National Institutes of Health, Bethesda, MD, USA). Lipid droplets were identified as unstained vacuole-like areas within the bone marrow cavity and manually counted from 15 representative microscopic fields per group under identical imaging conditions. Data are presented as mean ± SEM.

Statistical analysis: Data are presented as mean ± SEM. Statistical comparisons were performed using one-way ANOVA followed by Tukey’s post hoc test (version 16, SPSS, Chicago, IL, USA). A value of *p* < 0.05 was considered statistically significant.

## 3. Results

**Figure 1:**
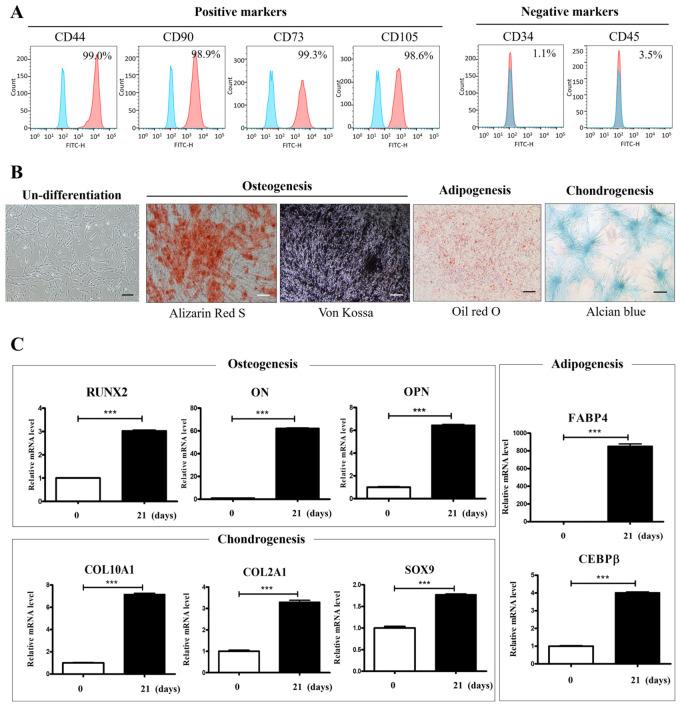
Phenotypic characterization and multilineage differentiation potential of dental tissue-derived MSCs. (**A**) Flow cytometric analysis of cell surface marker expression in D-MSCs at passage 3. D-MSCs were positive for the MSC-associated markers CD44, CD90, CD73, and CD105, whereas the hematopoietic markers CD34 and CD45 were not detected. Blue peaks indicate isotype IgG controls, and red peaks indicate expression of the corresponding CD markers. Data are representative of 3 biological replicates. (**B**) Histochemical evaluation of osteogenic, adipogenic, and chondrogenic differentiation of D-MSCs after 21 days of lineage-specific induction. Osteogenic differentiation was confirmed by Alizarin Red S and Von Kossa staining for calcium deposition and mineralization (×40, scale bar = 500 μm). Adipogenic differentiation was confirmed by Oil Red O staining of intracellular lipid droplets (×100, scale bar = 100 μm). Chondrogenic differentiation was confirmed by Alcian Blue staining of proteoglycans (×40, scale bar = 300 μm). (**C**) qRT-PCR analysis of lineage-specific gene expression following osteogenic, adipogenic, and chondrogenic differentiation. The (***) indicates a significant difference (*p* < 0.001) in mRNA expression between differentiated and undifferentiated cells. Data were obtained from 3 replicates.

*Isolation and Phenotypic Characterization of Dental Follicle-Derived MSCs*: MSCs were successfully isolated from human dental follicle tissue and expanded under standard culture conditions. The isolated cells exhibited plastic adherence and a fibroblast-like morphology with elongated spindle-shaped structures at passage 3, consistent with typical mesenchymal stem cell characteristics. Flow cytometric analysis further confirmed the mesenchymal phenotype. The cells showed positive expression of MSC-associated surface markers CD44, CD90, CD73, and CD105, while hematopoietic markers CD34 and CD45 were not detected (Figure 1A). These findings confirm that the isolated dental follicle-derived cells meet the established phenotypic criteria for MSCs.

*D-MSCs Exhibit Multilineage Differentiation Potential*: The multilineage differentiation capacity of D-MSCs was evaluated using lineage-specific histochemical staining and gene expression analysis (Figure 1B,C). Osteogenic differentiation was evidenced by the formation of mineralized nodules and calcium deposition, as demonstrated by positive Alizarin Red S and Von Kossa staining (Figure 1B). This was accompanied by a significant upregulation of osteogenesis-related genes *RUNX2*, *ON*, and *OPN* in differentiated cells compared with undifferentiated controls (*p* < 0.001) (Figure 1C). Adipogenic differentiation was confirmed by the accumulation of intracellular lipid droplets visualized by Oil Red O staining (Figure 1B), together with significantly increased expression of adipogenesis-related genes *FABP4* and *CEBPβ* (*p* < 0.001) (Figure 1C). Chondrogenic differentiation was demonstrated by proteoglycan deposition detected using Alcian Blue staining (Figure 1B), along with significantly elevated expression of chondrogenesis-related genes *COL10A1*, *COL2A1*, and *SOX9* (*p* < 0.001) (Figure 1C).

**Figure 2:**
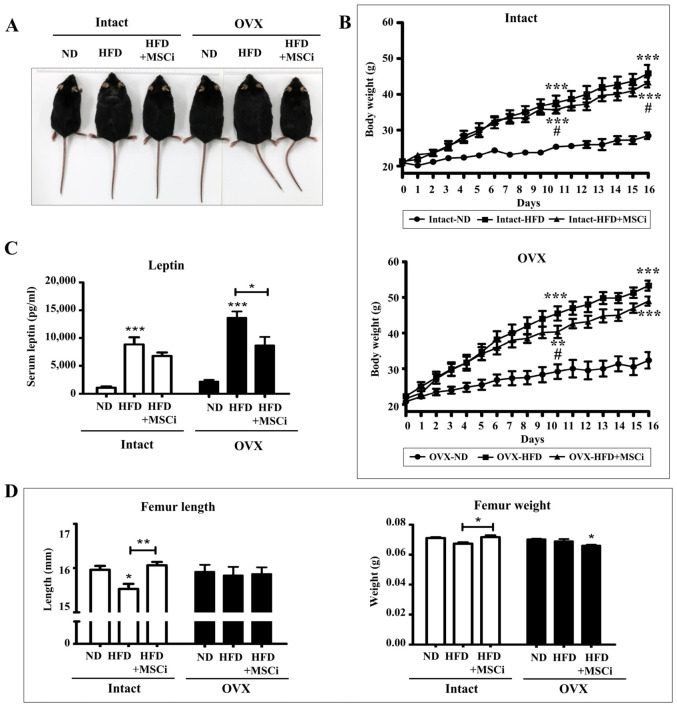
Effects of high-fat diet and MSC treatment on body weight, serum leptin levels, and femoral morphology in intact and ovariectomized mice. (**A**) Representative images of mice in each experimental group (intact-ND, intact-HFD, intact-HFD+MSCi, OVX-ND, OVX-HFD, and OVX-HFD+MSCi) at 16 weeks after diet initiation. (**B**) Changes in body weight in intact and OVX groups over the 16-week experimental period. Data are presented as mean ± SEM. For comparisons versus ND, ** *p* < 0.01 and *** *p* < 0.001; for comparisons versus HFD, # *p* < 0.05. (**C**) Serum leptin levels in each experimental group. Serum adipokine leptin was measured by ELISA. Data are presented as mean ± SEM. Statistical significance was determined by one-way ANOVA followed by Tukey’s post hoc test (* *p* < 0.05, *** *p* < 0.001). Data were obtained from 5–6 biological replicates with technical duplicates. (**D**) Morphometric analysis of femurs in each experimental group. Femur weight and femur length were compared among groups. Data are presented as mean ± SEM. Statistical significance was determined by one-way ANOVA followed by Tukey’s post hoc test (* *p* < 0.05, ** *p* < 0.01). Data were obtained from 5–6 biological replicates. OVX, ovariectomized; ND, normal diet; HFD, high-fat diet; HFD+MSCi, high-fat diet plus MSC injection.

*Establishment of an Obesity-Associated Osteoporosis Model and Effects of MSC Treatment on Body Weight*: An obesity-associated osteoporosis model was established by high-fat diet (HFD) feeding for 16 weeks in both intact and ovariectomized (OVX) mice. Representative images demonstrated clear differences in body weight among experimental groups at 16 weeks (Figure 2A). Body weight increased progressively in HFD-fed mice compared with normal diet (ND) controls under both intact and OVX conditions (Figure 2B). In intact mice, body weight was significantly higher in the HFD group than in the ND group (*p* < 0.001), whereas MSC treatment significantly reduced body weight at 5 and 16 weeks (*p* < 0.05). Similarly, OVX-HFD mice showed significantly increased body weight compared with OVX-ND mice (*p* < 0.001). MSC administration resulted in a significant reduction in body weight at 5 weeks (*p* < 0.05), although the reduction at 16 weeks did not reach statistical significance.

*MSC Treatment Modulates Serum Leptin Levels*: Serum leptin levels were significantly elevated in HFD groups under both intact and OVX conditions (*p* < 0.001) (Figure 2C). MSC treatment resulted in a modest, non-significant decrease in leptin levels in intact mice. In contrast, leptin levels were significantly reduced in the OVX-HFD+MSCi group compared with the OVX-HFD group (*p* < 0.05).

**Figure 3:**
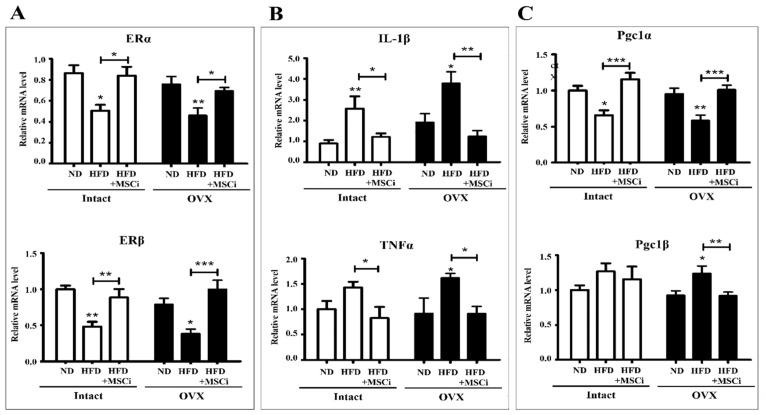
Effects of high-fat diet and MSC treatment on femoral expression of estrogen receptor-, inflammation-, and mitochondria-related genes. (**A**) qRT-PCR analysis of estrogen receptor expression (*ERα* and *ERβ*) in femoral tissues from each experimental group. (**B**) qRT-PCR analysis of inflammation-related genes (*Il-1β* and *Tnf-α*) in femoral tissues from each experimental group. (**C**) qRT-PCR analysis of mitochondria-related genes (*Pgc1α* and *Pgc1β*) in femoral tissues from each experimental group. For all panels, data are presented as mean ± SEM. Statistical significance was determined by one-way ANOVA followed by Tukey’s post hoc test (* *p* < 0.05, ** *p* < 0.01, *** *p* < 0.001). Data were obtained from 5–6 biological replicates with technical triplicates. OVX, ovariectomized; ND, normal diet; HFD, high-fat diet; HFD + MSCi, high-fat diet plus MSC injection.

*MSC Treatment Restores Femoral Morphology and Estrogen Receptor Expression*: Morphological assessment revealed that femur length was significantly decreased in the intact-HFD group (*p* < 0.05) and increased following MSC treatment (*p* < 0.01) (Figure 2D). In OVX mice, femur weight was slightly reduced in the HFD group and significantly increased after MSC treatment (*p* < 0.05), although it remained lower than in the OVX-ND group. No significant differences in femur length were observed among OVX groups. At the molecular level, qRT-PCR analysis demonstrated that *ERα* expression was significantly reduced in HFD groups under both intact and OVX conditions (*p* < 0.05–0.01) and significantly increased following MSC treatment (*p* < 0.05) (Figure 3A). Similarly, *ERβ* expression was significantly decreased in HFD groups (*p* < 0.05–0.01) and markedly restored in MSC-treated groups (*p* < 0.01–0.001) (Figure 3A).

*MSC Treatment Suppresses Inflammation-Related Gene Expression*: Expression of the pro-inflammatory cytokine *Il-1β* was significantly increased in both intact-HFD and OVX-HFD groups compared with their respective ND controls (*p* < 0.05–0.01) (Figure 3B). MSC treatment significantly reduced *Il-1β* expression in both intact and OVX conditions (*p* < 0.05–0.01). *Tnf-α* expression showed a slight increase in intact-HFD mice and a significant increase in OVX-HFD mice (*p* < 0.05). MSC administration significantly decreased *Tnf-α* expression in both intact and OVX groups (*p* < 0.05) (Figure 3B).

*MSC Treatment Regulates Mitochondrial Gene Expression*: *Pgc1α* expression was significantly reduced in HFD groups under both intact and OVX conditions (*p* < 0.05–0.01) and significantly increased following MSC treatment (*p* < 0.001) (Figure 3C). In contrast, *Pgc1β* expression was slightly elevated in intact-HFD mice and significantly reduced in the OVX-HFD+MSCi group compared with OVX-HFD mice (*p* < 0.01) (Figure 3C).

**Figure 4:**
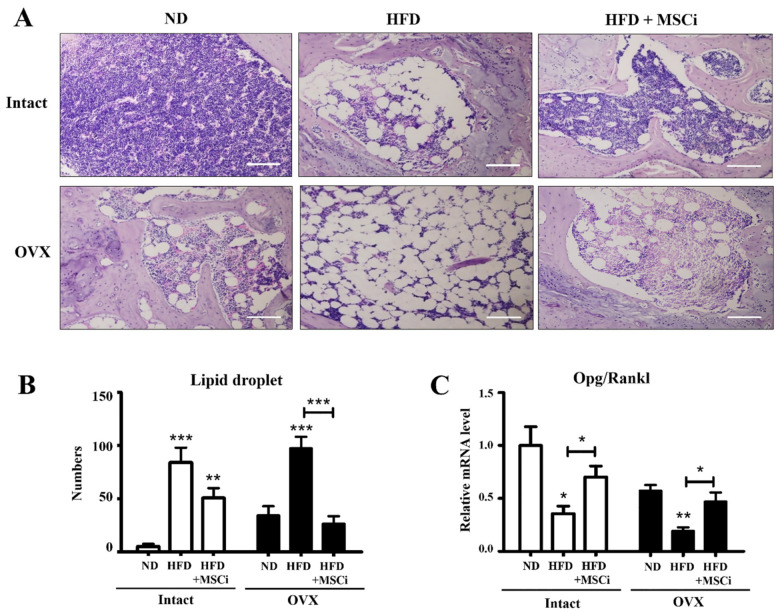
Histological assessment of marrow adiposity and analysis of the *OPG/RANKL* ratio in femoral tissues from the animal model. (**A**) Representative hematoxylin and eosin (H&E)-stained sections of femoral tissues from each experimental group (×100, scale bar = 200 μm). (**B**) Quantification of lipid droplet numbers in bone marrow areas. Each value was obtained from 15 images within the same group. (**C**) qRT-PCR analysis of the *OPG/RANKL* mRNA expression ratio in femoral tissues from each experimental group. Data were obtained from 5–6 biological replicates with technical triplicates. For quantitative analyses, data are presented as mean ± SEM. Statistical significance was determined by one-way ANOVA followed by Tukey’s post hoc test (* *p* < 0.05, ** *p* < 0.01, *** *p* < 0.001). OVX, ovariectomized; ND, normal diet; HFD, high-fat diet; HFD + MSCi, high-fat diet plus MSC injection.

*MSC Treatment Reduces Bone Marrow Adiposity*: Histological analysis using hematoxylin and eosin staining revealed increased fat accumulation within the bone marrow in intact-HFD, OVX-HFD, and OVX-ND groups (Figure 4A). This marrow adiposity was reduced in MSC-treated groups. Quantitative analysis showed that lipid droplet numbers were significantly increased in intact-HFD and OVX-HFD groups (*p* < 0.001). MSC treatment slightly reduced lipid droplets in intact mice and significantly decreased lipid droplet numbers in OVX mice (*p* < 0.001) (Figure 4B).

*MSC Treatment Restores the OPG/RANKL Balance*: The *OPG/RANKL* ratio was significantly decreased in HFD groups under both intact and OVX conditions (*p* < 0.05–0.01) (Figure 4C). MSC treatment significantly increased the *OPG/RANKL* ratio in all HFD + MSCi groups (*p* < 0.05), indicating restoration of bone remodeling balance.

## 4. Discussion

Obesity and menopause are two major conditions that reshape bone metabolism through intertwined endocrine and inflammatory mechanisms. Menopause-associated estrogen deficiency has a clear etiological relationship with osteoporosis; however, the role of obesity in skeletal homeostasis remains controversial. Increased body mass has traditionally been considered protective, partly through mechanical loading that stimulates bone formation [27], and adipocyte-derived estrogen has been proposed to inhibit osteoclast-mediated bone resorption [28]. In contrast, recent studies indicate that obesity-associated estrogen does not recapitulate ovarian estrogen signaling and may instead contribute to osteoporosis due to enhanced systemic inflammatory activity [29,30]. Against this background, the present study addressed whether dietary obesity induces osteoporotic alterations under both intact and estrogen-deficient conditions and evaluated the effect of D-MSC administration on obesity-associated skeletal dysregulation.

To model menopausal status, ovariectomy was employed, which is widely used and validated for osteoporosis induction in experimental settings [31,32,33,34]. Dietary obesity was induced using high-fat diet feeding, a common approach for establishing obesity without necessarily initiating disease-specific interventions. Previous work has reported that 16 weeks of high-fat diet feeding produces severe obesity accompanied by adipocyte hyperplasia, fat deposition, diabetes, hypertension, and brain damage in female mice [35,36]. In accordance with these reports, body weight increased markedly during 16 weeks of high-fat diet feeding, with maximal body weight of the HFD groups more than twice that of the ND group at the 16th week, and weight gain was higher in OVX mice than in intact mice. Consistent with the body weight data, leptin—a representative adipokine secreted by adipocytes and an index of obesity [37]—was increased in the HFD group and decreased upon MSC injection. These findings support that the experimental paradigm produced a robust obese phenotype in both intact and OVX females, and that MSC administration was associated with suppression of obesity induction as reflected by body weight and leptin measurements.

A key objective was to determine whether obesity induces skeletal changes indicative of impaired bone homeostasis under both menopausal and non-menopausal conditions. Direct changes in bone shape can be assessed by femoral length and weight measurements, and a previous study showed that both femoral length and weight were significantly reduced in osteoporosis-induced animals [38]. In the present study, femoral length was significantly reduced in the intact-HFD group and increased in MSC-administered intact animals, and femoral weight was increased in the OVX-HFD + MSCi group. At the same time, no significant differences in femoral length and weight were observed among OVX groups. These findings indicate that obesity was accompanied by detectable morphometric changes in the intact condition, while OVX conditions showed a distinct pattern. Importantly, measurement using Vernier calipers is not completely accurate and femur length and weight alone cannot determine whether osteoporosis is present. Accordingly, the present study emphasized a multi-layered assessment incorporating molecular remodeling and histological marrow adiposity rather than relying exclusively on gross morphometry.

At the level of estrogen signaling, obesity was associated with a reduction in estrogen receptor expression within femoral tissue. Both *ERα* and *ERβ* mRNA levels declined in all HFD groups and were restored in all MSC-treated groups. These results align with the concept that obesity-associated osteoporosis is not explained solely by circulating estrogen production from adipose tissue [28,29,30], but may also involve diminished estrogen responsiveness at the receptor level in bone. In parallel, inflammatory mediators *Il-1β* and *Tnf-α* were elevated in all HFD groups and reduced in all MSC-treated groups. Taken together, the combined reduction in estrogen receptor expression and elevation in inflammatory gene expression support an obesity-associated microenvironment in femoral tissue that favors bone resorption and disrupts remodeling balance; these changes were alleviated upon MSC injection.

Mitochondrial regulation was further evaluated as a mechanistic axis linking metabolic stress to bone remodeling. *Pgc1α* is a master regulator of mitochondrial biogenesis and inhibits skeletal stem cell differentiation into adipocytes while accelerating osteoblast differentiation via transactivation of Taz expression [13]. In contrast, *Pgc1β* regulates mitochondria in osteoclasts; *Pgc1β*-deficient osteoclast progenitors exhibit diminished mitochondrial biogenesis and impaired resorptive function [14]. In the present study, *Pgc1α* expression was lower in HFD groups than in ND groups and increased in MSC-treated groups. Conversely, *Pgc1β* was higher in HFD groups than ND groups and decreased in MSC-treated groups. This reciprocal pattern is consistent with obesity-associated suppression of osteoblast-linked mitochondrial biogenesis programs and enhancement of osteoclast-associated mitochondrial regulation, with reversal following MSC administration. In the present study, mitochondrial regulation was assessed indirectly by measuring the expression of mitochondrial regulatory genes, including Pgc1α and Pgc1β, in femoral tissue. Therefore, although D-MSC administration was associated with normalization of mitochondrial regulatory gene expression, the present data do not determine whether this effect resulted from direct mitochondrial transfer from D-MSCs to bone-resident cells or from indirect stimulation of endogenous mitochondrial recovery. However, direct confirmation of mitochondrial transfer requires additional experimental evidence, including mitochondrial labeling, cell tracking, confocal co-localization analysis, extracellular vesicle characterization, or inhibition of mitochondrial transfer pathways. Because these approaches were not included in the present study, direct mitochondrial transfer cannot be concluded in this model.

Instead, the reduced expression of inflammatory mediators, including Il-1β and Tnf-α, together with the restoration of Pgc1α and Pgc1β expression suggests that D-MSCs may have improved the inflammatory and metabolic status of the femoral microenvironment, thereby promoting endogenous recovery of mitochondrial regulatory pathways. This interpretation is consistent with the established roles of PGC-1α in skeletal stem cell fate, bone–fat balance, and mitochondrial regulation in bone homeostasis. Thus, the beneficial effects of D-MSCs observed in this study may involve coordinated paracrine, immunomodulatory, and mitochondrial regulatory mechanisms rather than a single direct mechanism.

Consistent with these molecular signatures, obesity altered the *OPG/RANKL* regulatory balance. RANKL binds to RANK on osteoclasts and promotes bone resorption [39], while OPG inhibits RANKL and restrains osteoclast activation, allowing bone formation to dominate over bone destruction [40]. The *OPG/RANKL* ratio directly correlates with bone formation [39]. In the present study, the *OPG/RANKL* ratio was decreased in HFD groups compared with ND groups and recovered in MSC-treated groups. Together with the ER, inflammatory, and mitochondrial findings, these data support a coherent remodeling imbalance in obesity—favoring osteoclastogenesis and suppressing osteoblastogenesis—followed by normalization toward ND-like patterns after MSC injection.

Histological analysis provided complementary tissue-level evidence. Osteoporotic mice have been reported to exhibit dominant bone marrow adiposity, trabecular bone spacing, and bone marrow cell depletion compared with healthy mice [41,42]. In the present study, increased fat accumulation in the bone marrow area was observed in both intact and OVX HFD groups, and the OVX-ND group also showed some marrow fat accumulation relative to intact-ND. Importantly, marrow fat accumulation appeared in remission in MSC-treated groups. Quantification further showed that the number of lipid droplets was significantly higher in intact-HFD and OVX-HFD groups, while lipid droplets were slightly decreased in intact-HFD + MSCi and significantly lower in OVX-HFD + MSCi compared with OVX-HFD. These findings are consistent with the concept that obesity and estrogen deficiency contribute to marrow adiposity, and that MSC treatment is associated with reduced marrow fat deposition.

The selection and characterization of the cellular product are central to interpreting therapeutic modulation of complex metabolic bone phenotypes. MSC-based cell therapies have emerged as a helpful strategy in regenerative medicine [43]. For therapeutic application, MSCs should be derived from young and healthy individuals and should be characterized by expression of MSC-specific cell surface markers and multilineage differentiation capacity [44]. In addition, selection of MSC sources with high osteogenic differentiation capacity is recommended for bone diseases. D-MSCs have the advantage of being obtained from unusable resources, demonstrate high expression of osteogenic-related genes including *RUNX2* and ALP [24], and exhibit robust immunomodulatory capacity due to continuous exposure to inflammatory conditions within the oral cavity [45,46]. In the present study, D-MSCs were successfully isolated from dental follicles of extracted wisdom teeth and displayed plastic adherence and fibroblastic morphology consistent with previous reports [47,48]. D-MSCs expressed MSC-associated markers (CD44, CD90, CD73, and CD50) and lacked hematopoietic markers (CD34 and CD45), consistent with bone marrow- and adipose-derived MSC profiles reported elsewhere [49,50]. Multilineage differentiation into osteogenic, adipogenic, and chondrogenic lineages was confirmed by lineage-specific staining and increased expression of lineage markers, consistent with prior demonstrations that D-MSCs possess strong osteogenic potency and have been applied to repair bone defects [51,52,53].

Several interpretive boundaries are relevant to framing these findings in a manner aligned with the experimental outputs. First, although femoral length and weight changes provide evidence of gross skeletal alteration, these metrics alone do not define osteoporosis and were explicitly interpreted in conjunction with molecular remodeling (ER signaling, inflammatory genes, mitochondrial regulators, and *OPG/RANKL* ratio) and marrow adiposity. Second, improvements in femoral molecular signatures occurred in parallel with attenuation of body weight gain and leptin levels, indicating that MSC administration was associated with systemic metabolic normalization alongside local femoral changes. Therefore, the observed skeletal improvements are interpreted as part of coordinated modulation of obesity-associated metabolic and remodeling dysregulation rather than as isolated direct engraftment effects. However, this study has a limitation in that it did not experimentally evaluate the direct mitochondrial delivery from D-MSCs to bone marrow-resident cells.

In summary, dietary obesity in female mice, under both intact and estrogen-deficient conditions, was associated with reduced estrogen receptor expression, increased inflammatory mediators, altered mitochondrial regulators, decreased *OPG/RANKL* ratio, and increased marrow adiposity in femoral tissue. D-MSC administration was associated with restoration of these parameters toward ND levels. These findings indicate that D-MSC treatment modulates coordinated metabolic and remodeling dysregulation in obesity-associated skeletal alteration irrespective of menopausal status.

## 5. Conclusions

In conclusion, dietary obesity in female mice was associated with coordinated alterations in femoral estrogen receptor expression, inflammatory mediators, mitochondrial regulatory genes, and the *OPG/RANKL* axis under both intact and estrogen-deficient conditions. These molecular changes were accompanied by increased marrow adiposity and morphometric alterations, indicating disruption of bone remodeling homeostasis. Administration of dental tissue-derived mesenchymal stem cells was associated with restoration of estrogen receptor expression, normalization of inflammatory and mitochondrial gene signatures, recovery of the *OPG/RANKL* ratio, and reduction in marrow fat accumulation. Collectively, these findings demonstrate that obesity induces bone remodeling dysregulation irrespective of menopausal status and that D-MSC treatment modulates this imbalance through coordinated regulation of inflammatory signaling, mitochondrial pathways, and osteoclast–osteoblast regulatory mechanisms. The present study provides mechanistic evidence supporting the application of D-MSCs in obesity-associated skeletal alteration under both pre- and postmenopausal conditions.

## Figures and Tables

**Table 1:** Antibodies for flow cytometry analysis to evaluate MSC-specific cell surface markers.

Antibody	Company	Amount	Dilution
FITC mouse anti-human CD34	BD Pharmingen^TM^	0.5 mg/mL	1:100
FITC mouse anti-human CD45	BD Pharmingen^TM^	0.5 mg/mL	1:100
FITC rat anti-human CD44	BD Pharmingen^TM^	0.5 mg/mL	1:100
FITC mouse anti-human CD90	BD Pharmingen^TM^	0.5 mg/mL	1:100
APC mouse anti-human CD73	BD Pharmingen^TM^	0.5 mg/mL	1:100
APC mouse anti-human CD105	BD Pharmingen^TM^	0.5 mg/mL	1:100

**Table 2:** qRT-PCR primer sequence for mouse tissues and human MSCs. (**a**) Primer sequence of estrogen receptors, inflammation-related genes, mitochondria-related genes, *Opg* and *Rankl* for mouse tissues. (**b**) Primer sequence of lineage-specific genes for human MSCs.

Target Gene	Sequence (5′–3′)	Product Size (bp)	Anneal. Tm. (°C)	Reference
(**a**)				
*Estrogen* *receptor-α*	F-AAGCGTCAGAGAGATGACTTGG R-CAGGGCTATTCTTCTTAGTGTGC	118	60	LC260510.1
*Estrogen* *receptor-β*	F-CAACTCGTTTCGCATTCCTACC R-AGTGACCACATTCAGACAGACC	184	60	NM_207707.1
*Il-1β*	F-ATGACCTGTTCTTTGAAGTTGACG R-CCTGAAGCTCTTGTTGATGTGC	128	60	BC011437.1
*Tnf-α*	F-ATGAGCACAGAAAGCATGATCC R-ATGAGAAGAGGCTGAGACATAGG	112	60	NM_001278601.1
*Pgc1α*	F-TCTTCCTTTAACTCTCCGTGTCG R-TGACCTGGAATATGGTGATCGG	138	60	BC066868.1
*Pgc1β*	*F*-GGACTGAGTTCTCTATCCTAAGGG *R*-GTGTGAGGGAAGCATAGACAGG	*102*	60	NM_133249.3
*OPG*	F-TGGACATCATTGAATGGACAAC R-TATAAGAGTGGTCAGGGCAAG	174	60	U94331.1
*RANKL*	F-CCGAGCTGGTGAAGAAATTAG R-TCTATGTCCTGAACTTTGAAAG	102	60	AF019048.1
*TBP*	F-AGTGAAGAACAATCCAGACTAG R-TATAGGGAACTTCACATCACA	129	60	NM_013684.3
(**b**)				
*RUNX2*	F-CTCTACTATGGCACTTCGTCAGG R-TTTAATAGCGTGCTGCCATTCG	119	60	NM_001015051.4
*ON*	F-GTGCAGAGGAAACCGAAGAG R-AAGTGGCAGGAAGAGTCGAA	130	60	NM_03118.2
*OPN*	F-TTGCAGCCTTCTCAGCCAA R-GGAGGCAAAAGCAAATCACTG	102	60	NM_001040058
*FABP4*	F-GGAAAGTCAAGAGCACCATAACC R-CATTCCACCACCAGTTTATCATCC	118	60	NM_001442.2
*CEBPβ*	F-TTTGTCCAAACCAACCGCACAT R-CAGAGGGAGAAGCAGAGAGTTTA	110	60	NM_001285879.1
*COL10A1*	*F*-AACAGGCAACAGCATTATGACC *R*-AAACATGAGTCCCTTTCACATGC	*103*	60	NM_000493.4
*COL2A2*	F-AGGAATTCGGTGTGGACATAGG R-GGAAAGTACTTGGGTCCTTTGG	100	60	NM_033150.2
*SOX9*	F-GACCTTTGGGCTGCCTTATATTG R-CTCCCTCACTCCAAGAGAAGATG	116	60	NM_000346.3
*YWHAZ*	F-CGAAGCTGAAGCAGGAGAAG R-TTTGTGGGACAGCATGGATG	111	60	NM_003406.3

## Data Availability

All primary data are included in the article figures. All original data are available upon reasonable request from the corresponding author.

## Abbreviations

The following abbreviations are used in this manuscript:

A-DMEM	Advanced Dulbecco’s Modified Eagle’s Medium
ALP	Alkaline phosphatase
D-MSCs	Dental tissue-derived mesenchymal stem cells
D-PBS	Dulbecco’s phosphate-buffered saline
ER	Estrogen receptor
ALP	Estrogen receptor alpha
D-MSCs	Estrogen receptor beta
D-PBS	Fetal bovine serum
ER	Fibroblast growth factor
*ERα*	High-fat diet
*ERβ*	Insulin-like growth factor-1
FBS	Interleukin-1 beta
FGF	Mesenchymal stem cells
HFD	Alkaline phosphatase
IGF1	Dental tissue-derived mesenchymal stem cells
*Il-1β*	Dulbecco’s phosphate-buffered saline
MSCs	Estrogen receptor
ND	Normal diet
OPG	Osteoprotegerin
OVX	Ovariectomized
*Pgc1α*	Peroxisome proliferator-activated receptor gamma coactivator-1 alpha
*Pgc1β*	Peroxisome proliferator-activated receptor gamma coactivator-1 beta
RANK	Receptor activator of nuclear factor-κB
RANKL	Receptor activator of nuclear factor-κB ligand
TGFβ	Transforming growth factor beta
*Tnf-α*	Tumor necrosis factor alpha
